# From decision to destination: factors influencing healthcare students’ career paths in Qatar

**DOI:** 10.1186/s12909-024-06527-3

**Published:** 2024-12-23

**Authors:** Yasin M. Yasin, Albara Alomari, Shannan MacNevin, Baysan Kmainasi, Mohammad Abdulla, Sondos Hassanin

**Affiliations:** 1 Nursing and Midwifery, College of Health Sciences, University of Doha for Science and Technology, P. O. Box 24449, Doha, Qatar; 2 Pharmacy Technology, College of Health Sciences, University of Doha for Science and Technology, Doha, Qatar; 3 Environmental Health, College of Health Sciences, University of Doha for Science and Technology, Doha, Qatar

**Keywords:** Healthcare education, Career path, Support, Facilitators, Challenges, Students

## Abstract

**Background:**

Healthcare education is crucial for building a workforce in Qatar that is in line with Vision 2030. Although various factors influence career choices, little research exists on their impact on healthcare students in Qatar. This study explores the factors influencing healthcare students in Qatar to pursue healthcare careers, as well as the facilitators and challenges they face during their education.

**Methods:**

A cross-sectional survey was conducted among 364 healthcare students in Qatar. Quantitative data was analyzed to identify key factors influencing career choice, while qualitative data provided deeper insights into challenges and support systems.

**Results:**

Quantitative findings identified “contribution to society” and “quality” as key factors shaping career choices. Demographic variables such as gender, income, and birthplace influenced these decisions. Qualitative analysis revealed that students face numerous challenges, such as academic demands, financial pressures, and logistical difficulties, but these were mitigated by support from family, peers, and institutional resources.

**Conclusion:**

This study underscores the importance of tailored support systems to foster student retention and success in healthcare education, particularly for students from diverse cultural backgrounds. Policymakers and educators should develop strategies that support a sustainable, culturally competent healthcare workforce in Qatar that meets the nation’s evolving healthcare needs.

**Clinical trial number:**

Not applicable.

**Supplementary Information:**

The online version contains supplementary material available at 10.1186/s12909-024-06527-3.

## Introduction

The global demand for skilled healthcare professionals continues to grow, driven by the evolving healthcare landscape and the diverse health needs of individuals and populations [1]. In Qatar, the Qatar National Vision 2030 emphasizes developing a knowledge-based economy with a focus on human development and sustainable growth [2]. Addressing workforce shortages in the healthcare sector, as seen in many Middle Eastern and North African countries, is central to achieving these goals [3].

Pursuing a healthcare career is a significant and often complex decision shaped by a combination of intrinsic, extrinsic, and interpersonal factors [4]. Research shows that intrinsic motivations, such as personal interest and the desire to contribute to society, play a primary role in healthcare career choices [1, 5]. Moreover, financial incentives, intellectual challenges, and opportunities for lifelong learning enhance the appeal of healthcare careers [6, 7]. For example, paramedic students are often motivated by a desire to help others and the excitement of saving lives [8], reflecting the broader values of altruism and societal contribution [9].

Extrinsic factors, including job availability and societal recognition, also play a vital role in healthcare career decision-making [9, 10]. The high regard for healthcare professionals enhances the appeal of these professions [11, 12]. Moreover, families, teachers, and peers are influential, particularly in collectivist cultures where professional status and societal expectations significantly shape career choices [1, 13].

Cultural context strongly influences career decisions. In collectivist cultures, societal prestige and alignment with family expectations often dictate career choices [14]. Conversely, individualistic cultures prioritize personal satisfaction and self-fulfillment, with young individuals gaining confidence from personal achievements rather than relying on familial or societal validation [14]. These cultural distinctions illustrate the complexity of factors influencing healthcare choices globally. Despite the growing demand for healthcare professionals [15, 16], limited research has explored the factors shaping healthcare career decisions in Qatar. This study aims to address this gap by examining the factors influencing undergraduate healthcare students’ career decisions and the facilitators and challenges they face in completing their education after making their healthcare career choices.

The study addressed three key research questions:

1. What factors influence the healthcare career choices of undergraduate students in Qatar?
2. How do personal, educational, and societal factors interact to shape healthcare career decisions among undergraduate students in Qatar?
3. What are the primary facilitators and challenges faced by undergraduate healthcare students in Qatar as they pursue their education after choosing a healthcare career?

This study enhances the understanding of career decision-making among healthcare students in Qatar by providing a comprehensive analysis of the factors influencing these decisions. It highlights the unique interplay of intrinsic motivations, extrinsic incentives, and cultural expectations that shape students’ choices. By identifying the specific facilitators and challenges encountered during their academic journey, this research provides practical insights for policymakers and educators to develop targeted support strategies. Additionally, the findings contribute to the global literature by situating Qatar’s experience within the broader context of healthcare education, enriching discussions on how cultural and socioeconomic factors influence career pathways.

## Theoretical framework

Several models have been developed to describe the factors influencing career choice. Among these, the expectancy-value model of career decision-making is widely recognized for its application in predicting students’ career choices [17]. A notable adaptation of this model is Watt and Richardson’s framework, which includes both the perceived abilities necessary for success and the capacity to navigate potential challenges in a given profession [18]. This model identifies key value components, including intrinsic value (e.g., interest in a specific career), personal utility value (e.g., job security), and social utility value (e.g., the desire to help others and contribute to society). It also considers external factors like social status, prior experiences, learning experiences, and persuasion by others as influential in shaping career decisions.

The Healthcare Career Choice Scale (HCCS) used in this study was derived from this model, aligning with its focus on intrinsic and extrinsic factors. Furthermore, Watt and Richardson’s framework guided the analysis of qualitative data, offering a robust lens for interpreting how students’ motivations and external influences interact to shape career decisions. By linking these motivations with external factors, this model adds depth to our findings. The insights gained will inform targeted strategies to attract and retain students in high-demand healthcare specialties, ultimately contributing to the development of a sustainable healthcare workforce in Qatar.

## Methods

A cross-sectional, descriptive correlational survey design was used to examine factors influencing healthcare career choices among students in Qatar. This design was chosen for its capacity to gather information at a single point in time, offering a cost-effective and efficient approach for examining associations between facilitators, challenges, and demographic differences without the need for longitudinal methods [19, 20]. The study utilized a combination of quantitative and qualitative data to address the complexity of healthcare students’ career decision-making. Quantitative data identified general trends and associations between demographic variables and career choice factors, providing generalizable insights. Meanwhile, qualitative data offered a deeper understanding of students’ motivations, barriers, and contextual challenges. This dual approach allowed us to address both the broad patterns and individual nuances of career decision-making, leading to a more thorough understanding of the phenomenon [21].

### Instruments

#### Demographic data

Demographic information was collected to provide a comprehensive understanding of the sample population. The data included variables such as age, gender, nationality, educational institution, sponsorship status, income, place of high school completion, healthcare specialty, years enrolled in the university, and high school grade averages. These variables were analyzed to identify associations with factors influencing healthcare career choices factors.

#### Healthcare career choice scale

In this study, we utilized the HCCS based on the Watt and Richardson’s framework, to explore the factors driving healthcare career choices among students [4]. The scale enabled us to measure the interplay between intrinsic value, personal utility value, social utility value, and external factors providing a comprehensive understanding of the motivations behind students’ decisions. By examining these factors, our research offers critical insights into the evolving healthcare workforce and contributes meaningfully to the broader discourse on developing a sustainable, responsive workforce in rapidly changing healthcare environments. The HCCS is a 43-item self-report questionnaire rated on a 5-point Likert scale, ranging from 1 (strongly disagree) to 5 (strongly agree), with higher scores indicating a greater influence of the factor on healthcare career choice [4]. Originally developed in Saudi Arabia, the HCCS has demonstrated high internal consistency (Cronbach’s α of 0.91), making it suitable for this study [4]. Factor analysis of the HCCS yielded 12 components that explained 71.8% of the total variance, including “contribution to society,” “social status,” “career value and perceived abilities,” “work with patients,” “satisfaction with choice,” “job security,” “prior experiences,” “qualities,” “social influences,” “fallback career,” “difficulty,” and “social dissuasion” [4]. Detailed descriptions of these components are available in the supplementary file.

#### Open-ended questions

Three open-ended questions were incorporated into the survey to explore the facilitators and challenges students faced after selecting a healthcare career. These questions provided an opportunity for participants to reflect on their personal experiences and elaborate on their motivations, barriers, and facilitators for success. The first question, “If you could go back in time, would you still choose your profession? And why?”, encouraged respondents to evaluate their satisfaction with their healthcare career choice and articulate the factors influencing their decisions. The second question, “What are the challenges faced in terms of academic demands, personal life, social pressures, or financial burdens during your study?”, allowed participants to highlight specific obstacles they encountered during their education. The last question, “What are the facilitators that helped you overcome the challenges during your studies?”, invited students to share insights into the resources, strategies, and support systems that enabled their success. These open-ended questions added depth to the study by capturing rich, qualitative data that complementing the quantitative findings.

### Data collection

The data collection took place at the University of Doha for Science and Technology (UDST), the only university in Qatar offering allied health programs. An online survey was distributed to all healthcare students within the College of Health Sciences (CHS). A non-random sampling method was used, targeting the entire CHS student population to maximize participation. This approach was chosen due to the accessibility of participants within the institution.

Potential participants received an email invitation with a survey link and detailed information about the study’s purpose, eligibility criteria, and voluntary nature. Students were eligible to participate if they [1] could read, understand, and speak English; [2] were 18 years of age or older; [3] were actively enrolled in an undergraduate healthcare specialty program; and [4] provided informed consent by completing the online survey. Ethical approval was obtained from the Primary Health Care Corporation Institutional Review Board (BUHOOTH-D-23-00088), ensuring ethical compliance.

A priori sample size calculations were performed via G*Power3 software [22]. Parameters included a two-tailed test, one-way ANOVA as the statistical test, a medium effect size (*F* = 0.25), six groups, and an alpha level of 0.05. On the basis of these parameters, the results indicated that a total sample of 324 participants was necessary to achieve a statistical power of 0.95. Data collection commenced in March 2024 and continued for three months. The initial survey was distributed to 800 students, resulting in a response rate of 45.5% (364 participants).

To address common method bias, several mitigation strategies were used, including ensuring anonymity in responses to reduce social desirability bias, emphasizing the voluntary nature of participation, and using Harman’s single-factor test to assess whether common method bias was present. Results from the test confirmed that common method bias did not significantly influence the findings.

### Data analysis

Quantitative data were analyzed using SPSS (version 29). Descriptive statistics, including means and standard deviations, were used to summarize the continuous variables, whereas categorical data were reported as counts and percentages. The factors related to healthcare career choices were described using means, standard deviations, and choice ratings specific to healthcare specialty programs. Associations between demographic variables and healthcare career choice factors were assessed using ANOVAs and *t*-tests, with statistical significance set at *p* < 0.05.

Qualitative data from open-ended questions were entered in an Excel spreadsheet. A content analysis approach was used to identify and organize emerging themes and sub-subthemes [23]. Two authors independently reviewed the data multiple times to identify emerging meaning units. These codes were grouped and given initial codes, aiding in the identification of categories and themes [23]. The coding and analysis process was iterative, with the research team discussing and refining the themes until a consensus was reached on both the final themes and the terminology used to describe them.

To ensure the rigor of the qualitative analysis, the authors adhered to the four pillars of trustworthiness in qualitative research: credibility, confirmability, dependability, and transferability [24]. Credibility was ensured through triangulation, comparing qualitative findings with quantitative data for cross-validation. Member checking was performed by approaching a subset of students who had participated in the original survey and were willing to identify themselves to the research team. These students were provided with a summary of the qualitative findings and asked to validate our interpretations. Peer debriefing among the research team further enhanced credibility. Transferability was supported by providing thorough and detailed descriptions of the research context and participant demographics, allowing others to assess the applicability of the findings in similar contexts. Confirmability was achieved through peer debriefing, ensuring that the interpretations were grounded in the data. Dependability was maintained by documenting the analytical process in detail, ensuring consistency and transparency throughout the research.

## Results

### Sample characteristics

A total of 364 healthcare students participated in the study, with 82.4% identifying as female. The majority (50.8%) were aged between 20 and 24 years, followed by students aged 20 years or younger (36.8%) and a smaller percentage aged 25 years or older (12.4%). Financial support was predominantly provided by families (88.7%), with the remainder relying on government sponsorship (5.8%), privately sponsorship (4.7%), or other sources (0.8%).

In terms of monthly income, nearly half of the students reported family incomes exceeding QAR 10,000 (49.2%), whereas those earning between QAR 5,000 and QAR10,000 accounted for 40.9% of the sample. A smaller percentage, 9.9%, reported incomes less than 5,000. The geographical origins of the students showed a slight majority born in Qatar (53.8%), compared to 46.2% born outside of Qatar. With respect to their high school education, a vast majority had attended school in Qatar (93.7%), whereas only 6.3% attended school abroad.

The high school performance distribution revealed that 33.2% of students scored above 90, 26.6% scored between 85 and 89, 23.4% scored between 80 and 84.9, 10.4% scored between 75 and 79.9, 4.7% scored between 70 and 74.9, and 1.6% scored between 65 and 69.9. Among healthcare specialties, respiratory therapy was the most common field of study (19.5%), followed by nursing and midwifery (17.6%), pharmacy (17.3%), medical radiography (16.2%), paramedicine (15.1%), and dental hygiene (11.5%). The enrollment durations indicated that 48.9% of the students had been enrolled for 3–4 years, 23.6% for 1–2 years, 17.6% for less than one year, and 9.9% for more than four years. In terms of career satisfaction, 70.6% of the students reported being satisfied with their choices, while 29.4% expressed a desire to switch careers. See Table 1 for more details.

**Table 1:** Sample characteristics

Characteristics		Total (***N*** = 364)	
		***n***	%
**Age**	20 or less	134	36.8
	20–24	185	50.8
	25 or more	45	12.4
**Gender**	Female	300	82.4
	Male	64	17.6
**Sponsorship**	Family	323	88.7
	Government	21	5.8
	Private	17	4.7
	Others	3	0.8
**Income QAR/month** ^*^	< 5,000	36	9.9
	5,000–10,000	149	40.9
	> 10,000	179	49.2
**Birth**	Outside Qatar	168	46.2
	Inside Qatar	196	53.8
**High school**	Outside Qatar	23	6.3
	Inside Qatar	341	93.7
**Enrollment duration**	< 1 year	64	17.6
	1–2 years	86	23.6
	3–4 years	178	48.9
	> 4 years	36	9.9
**High school average**	65-69.9	6	1.6
	70-74.9	17	4.7
	75-79.9	38	10.4
	80-84.9	85	23.4
	85–89	97	26.6
	90 or more	121	33.2
**Specialty**	Dental Hygiene	42	11.5
	Radiography	59	16.2
	Nursing & Midwifery	64	17.6
	Paramedicine	55	15.1
	Pharmacy	63	17.3
	Respiratory	71	19.5
**Career choice satisfaction**	No	107	29.4
	Yes	257	70.6

^* 1 QAR= 0.274 US Dollar^

### Factors affecting students’ decision to pursue a healthcare career

The factors most strongly associated with healthcare career decisions were “Contribution to Society” at 4.32 (*SD* = 0.748) and “Quality” of the healthcare profession at 4.24 (*SD* = 0.907). Moderately influential factors included “Career Value and Perceived Abilities” at 3.99 (*SD* = 0.818), “Satisfaction with Choice” at 3.96 (*SD* = 1.044), “Work with Patients” at 3.92 (*SD* = 1.068), “Job Security” at 3.72 (*SD* = 0.923), and “Prior Experience” at 3.81 (*SD* = 1.070). Lower-impact factors were “Social Status” at 3.68 (*SD* = 0.973), “Social Influence” at 3.25 (*SD* = 1.096), and “Difficulty” at 3.51 (*SD* = 1.102). The least influential factors were “Fallback Career” 2.91 (*SD* = 1.246) and “Social Dissuasion” 2.84 (*SD* = 1.243), indicating minimal impact on student decisions and high variability in response. These findings highlight the complex interplay of personal, social, and economic considerations in shaping healthcare career decisions. See Table 2 for more details.

**Table 2:** Factors affecting students’ decision to pursue a healthcare career

	Mean^±^	SD
**Contribution to society**	4.32	0.748
**Social status**	3.68	0.973
**Career value and perceived abilities**	3.99	0.818
**Work with Patients**	3.92	1.068
**Satisfaction with choice**	3.96	1.044
**Job Security**	3.72	0.923
**Prior Experience**	3.81	1.070
**Quality**	4.24	0.907
**Social Influence**	3.25	1.096
**Fallback Career**	2.91	1.246
**Difficulty**	3.51	1.102
**Social Dissuasion**	2.84	1.243

± Scores range between 1 and 5

### Demographic and socioeconomic influences on healthcare career choice factors

Demographic and socioeconomic factors significantly influenced healthcare career choices in several domains. Notably, students born outside Qatar expressed less interest in working with patients compared to those born inside Qatar, *t*(336.59) = -0.244, *p* < 0.01. No other demographic or socioeconomic variables had a statistically significant impact on this career choice factor.

Job security significantly differed by gender and birth location. Male students and those born outside Qatar placed greater importance on job security compared to females and students born inside Qatar, *t*(102.45) = -2.58 and *t*(361.02) = 2.04, respectively, *p* < 0.05. Among healthcare specialties, dental hygiene students placed greater value on job security compared to pharmacy students, *F*(5, 160.44) = 3.24, *p* < 0.01. No other demographic or socioeconomic factors had a significant effect on job security as a factor in healthcare career choice.

Family income also influenced healthcare career decision-making. Students from higher-income families (above QR10,000) valued “Quality” more than those from middle-income families (QR5,000 and QR10,000) did, *F*(2, 94.73) = 5.67, *p* < 0.01. “Social Influence” showed a gender disparity, with males perceiving greater social influence on their career choices compared to females, *t*(99.88) = -2.20, *p* < 0.05). “Social Dissuasion” significantly differed by healthcare specialty, *F*(5, 158.52) = 4.35, *p* < 0.001, with respiratory therapy students reporting less impact from social dissuasion than students in nursing and midwifery, paramedicine, medical radiography, and pharmacy programs. No other demographic or socioeconomic factors significantly influenced the variables “Quality”, “Social Influence”, or “Social Dissuasion”. Other factors, including “Contribution to Society”, “Social Status”, “Career Value and Perceived Abilities”, “Satisfaction with Choice”, “Prior Experience”, “Fallback Career”, and “Difficulty,” did not show statistically significant differences across age, gender, income, birth location, high school location, or healthcare specialty, with *p* values well above 0.05. See Table 3 for more details.

**Table 3:** Demographic and socioeconomic influences on healthcare career choice factors

Factors affecting career choice	Age		Gender		Income		Birth in Qatar		Place of high school		Healthcare specialty	
	*F*	*df1/df2*	*t*	*df*	*F*	*df1/df2*	*t*	*df*	*t*	*df*	*F*	*df1/df2*
**Contribution to society**	2.31	2/112.60	0.76	86.91	1.43	2/106.03	0.26	360.99	− 0.57	26.59	1.01	5/159.15
**Social status**	0.05	2/118.70	-1.80	106.49	1.65	2/99.72	0.76	361.62	0.86	24.42	0.88	5/158.84
**Career value and perceived abilities**	0.11	2/115.21	0.31	94.68	1.85	2/96.89	− 0.095	357.09	0.46	24.73	1.25	5/160.77
**Work with Patients**	0.13	2/120.51	1.67	96.32	1.26	2/98.87	-2.44^**^	336.59	− 0.30	25.23	1.14	5/160.37
**Satisfaction with choice**	0.21	2/123.66	-1.18	104.98	2.43	2/97.22	0.46	356.02	0.05	24.59	1.35	5/160.48
**Job Security**	1.19	2/117.65	-2.58^*^	102.45	0.33	2/105.86	2.04^*^	361.02	0.13	26.58	3.24^**^	5/160.44
**Prior Experience**	1.55	2/128.10	-1.27	107.67	2.72	2/99.98	0.33	354.34	1.58	26.38	2.07	5/159.47
**Quality**	0.33	2/118.94	− 0.27	91.07	5.67^**^	2/94.73	0.85	352.78	0.28	28.39	1.74	5/158.84
**Social Influence**	2.58	2/120.40	-2.20^*^	99.88	0.88	2/99.98	-1.05	359.79	0.344	25.54	1.95	5/159.40
**Fallback Career**	3.04	2/126.25	1.722	91.799	1.89	2/95.95	-1.54	341.08	− 0.06	24.18	1.11	5/158.38
**Difficulty**	3.03	2/120.16	0.50	86.09	0.13	2/96.16	0.56	335.53	-1.16	24.24	1.60	5/158.96
**Social Dissuasion**	2.51	2/132.65	1.13	96.13	1.77	2/96.92	− 0.58	351.14	0.85	24.49	4.35^***^	5158.52

*=*p* < 0.05, **=*p* < 0.01, ***=*p* < 0.001

### Qualitative results

The qualitative data revealed three main themes: Multifaceted Motivations in Healthcare Specialty Selection, Navigating Complex Challenges in Healthcare Education, and Diverse Facilitators Supporting Healthcare Education. See Table 4 for qualitative findings.

**Table 4:** Qualitative findings

Themes	Sub-Themes	Examples
**Multifaceted Motivations in Healthcare Specialty Selection**	Intrinsic Value (Interest in Healthcare)	Personal passion for helping others; Long-standing interest in healthcare (e.g., childhood dreams)
	Personal Utility Value (Job Security and Career Prospects)	Stability and demand in the job market (e.g., during crises like COVID-19); Career prospects (work hours, compensation)
	Social Utility Value (Desire for Social Contribution)	Desire to make a positive impact on society; Sense of purpose in serving others
	External and Circumstantial Factors	Limited options during registration; Influence of personal/family health experiences; External factors (social status)
**Navigating Complex Challenges in Healthcare Education**	Academic Demands	Heavy coursework, clinical rotations; Anxiety related to academic performance
	Emotional and Physical Exhaustion	Exhaustion from clinical rotations; Psychological pressure from studies and exams
	Balancing Personal Life	Difficulty balancing rigorous program with personal/social life
	Logistical and Financial Challenges	Commuting long distances; Financial burdens (tuition, materials, scholarships)
**Diverse Facilitators Supporting Healthcare Education**	Institutional Support	Academic support services (e.g., peer tutoring, help centers, instructors); Faculty and academic advisors
	Personal Strategies	Internal motivation and learning techniques (flashcards, YouTube, practice exams)
	External Resources	Online learning platforms (YouTube, professor visits)
	Social Support	Emotional and financial support from family and friends; Peer support from fellow students

#### Multifaceted motivations in healthcare specialty selection

This theme reflects the varied and interwoven reasons students provided for choosing specific healthcare fields. These reasons ranged from personal passions to practical and circumstantial factors. Students’ healthcare specialty selection was shaped by a rich tapestry of intrinsic motivations, such as a desire to help others and personal interests in particular medical fields, as well as extrinsic considerations, such as job security, market demand, and practical aspects such as work hours and compensation.

Approximately 25% of the students expressed a deep-rooted passion for healthcare, often connected to a desire to help others or a long-standing interest in the field. One respondent noted, “*It was my childhood dream to be a nurse*,* and I feel I’m in the right profession*,* because I like to help people and deal with them.*” Another respondent wrote, “*It was the profession I was thinking about since I was a high school student*.”

Conversely, 21% of the students reported choosing their healthcare specialty due to the limited options available during their registration rather than a strong initial preference. This is reflected in comments such as “*Was the only specialty that was open during my enrollments*”, “*It was my third choice*,* it was the last resort course for me”*, and “*It was the only bachelor’s degree in health science that was offered at the time I applied.*” Another student noted: “*It was either this or Nursing*,” indicating that the choice was largely influenced by what was immediately accessible.

Many respondents (18%) highlighted perceived stability and demand in the job market as significant factors in their decision-making process. For example, students emphasized the ongoing need for healthcare professionals during crises, such as the COVID-19 pandemic: “*in terms of job security*,* most importantly in times of crisis like the COVID-19 pandemic*,* wherein a shortage for nurses exists*.” Others mentioned the appeal of good career prospects: “*It has a good career path and a guaranteed demand in the market.*” Practical considerations such as work hours, compensation, and job conditions were also identified by respondents (11%). One student noted, “*Chose this program as it’s in health sciences and doesn’t require much patient interaction. Reasonable working hours*,* good pay*,* good career.*”

Personal or family health experiences significantly influenced the specialty choices of 11% of the students. One student, for example, related their choice to their own medical history: “*The reason why I chose this career path is because of my experience of when I was a patient at the ages 7 to 9 years old in my childhood. I had a heart condition that needed to be treated via surgery.*” Another participant commented that I was influenced by family experiences in healthcare: “*I have chosen this program because I have future visions and I took it because I like it*,* influenced by family members in the field.*”

#### Navigating complex challenges in healthcare education

Healthcare education presents a myriad of challenges that affect students across academic, personal, and financial dimensions, all of which shape the overall educational experience. The students shared insights into these difficulties, revealing the resilience required to navigate the demands of healthcare education.

Approximately 43% of the students referred to the heavy academic demands of their programs, which often included extensive coursework, clinical rotations, and high expectations. One student stated, “*A lot of information*,* high expectations*,” whereas another stated the difficulty of balancing “*rigorous coursework*,* extensive lab sessions*,* and demanding clinical placements.*” Several students expressed anxiety related to academic performance. One noted, “*Very anxious due to the 70% overall grade. Seeing other batches failing courses and changing into a different program is very stressful*,” whereas another student said, “*Academically*,* there were too many changes in our plans which made me consider dropping*.”

Physical and emotional exhaustion was frequently associated with clinical rotation and practical requirements. One student described: “*Clinical rotations can be draining*,” whereas another commented on the “*psychological pressure when studying and during exams.”* Balancing the demands of a rigorous program with personal and social life was challenging for many, with one student stating, “*It can get a bit stressful and it’s hard to have time to myself.*”

Additionally, 35% of the students reported logistical challenges, such as commuting long distances to campus. One student noted, “*My home is far from the university*,* which is a struggle since I do commute.*” Financial burdens were also a common theme, with high tuition fees, the cost of materials, and the need to maintain scholarships posing difficulties. One student mentioned, “*I lost my scholarship so I’m finding a lot of difficulties I hope I get it back*,* I increased my GPA*.” Another added, “*Financial burdens are my number one challenge in terms of studying abroad.*”

#### Diverse facilitators supporting healthcare education

To navigate these challenges, students relied on various facilitators, including institutional support systems, personal strategies, and social networks. The responses underscore the critical role of these facilitators in enabling students to navigate academic, personal, and professional demands effectively.

Institutional resources were essential for approximately 31% of the students. One student highlighted the availability of *“peer tutoring*,” while another praised the academic support services: “*Help centers and availability of my instructors at the times I needed them*.” Faculty and academic advisors also played supportive roles. One respondent noted, “*Fortunately*,* all of the pharmacy instructors are very helpful and explain everything well*,” and another acknowledged the assistance of “*Advisors and instructors who are always supportive*.”

For some students, internal motivation and strategic learning methods were crucial to managing academic demands. Approximately 15% of the students described using techniques such as “*Reviewers and flashcards*” and “*watching YouTube*” to cope with academic demands. Online platforms and external resources were used extensively by 12% of the students for supplementary learning tools. Statements such as “*videos on sites like YouTube were also useful*” and “*I use YouTube videos*,* or I’ll visit the course professor to help me understand*” highlight the reliance on digital tools. Additionally, practical tools such as “*practice exams before the actual exam*” and innovative study techniques help individuals cope with the rigorous academic workload.

Emotional and, in some cases, financial support from family and friends is vital. Approximately 42% of the students expressed gratitude for their support systems, stating, “*My family*” and “*My friends*,* family*,* and dedicated instructors*.” Peer support, particularly from fellow students in the same field, provides both academic and moral support. This is encapsulated by *“student friends in the same major*” and *“friends helped me understand the courses better.*”

## Discussion

This study provides valuable insights into the factors influencing healthcare career choices, as well as the facilitators and challenges that students face throughout their education in Qatar. The demographics reflect a predominantly young, female student body, with most relying on familial financial support. This suggests a cultural inclination toward family-supported education in healthcare fields [25] emphasizing the role of family dynamics in shaping educational pursuits. The interplay of intrinsic and extrinsic motivations significantly influence career decisions, aligning with literature that emphasizes both altruistic and pragmatic factors in healthcare career choices [14].

### Career choice

Quantitative findings identified “Contribution to Society” and “Quality” as the most influential factors driving students’ decisions to pursue healthcare careers. Qualitative findings supported these results, with many students expressing a deep-rooted passion for healthcare and a desire to help others and contribute to societal well-being. Altruism, a core value in healthcare professions, motivates many to pursue a healthcare career path [26]. Additionally, the deep-rooted passion for healthcare, connected to helping others and a sustained interest in the field, highlights the importance of intrinsic motivators over extrinsic rewards like financial gain [27]. Personal or family health experiences further shaped career preferences, corroborating the moderate influence of prior experience on career decisions observed in quantitative data.

Interestingly, a notable portion of students chose their healthcare specialty based on circumstantial factors, such as limited registration options. This finding aligns with the lower influence for factors like “Social Status” and “Fallback Career.” Although some students initially chose their specialty by default, students continued to value the profession’s core attributes, such as job security and societal contribution. Research suggests that constraints can affect not only immediate educational outcomes but also long-term job satisfaction and career development within the healthcare sector [28, 29]. For example, a mismatch between passion and specialty can lead to lower job satisfaction and higher burnout rates [30].

The quantitative data also revealed demographic and socioeconomic influences on career choices. For example, students born outside Qatar, primarily from expatriate families, showed less interest in patient interaction. Additionally, male students, those born outside Qatar, and students in certain specialties prioritized job security. These findings are consistent with the qualitative data, where expatriates, in particular, emphasized job security, likely due to visa dependencies and temporary work contracts [31, 32]. This need for security could be more pronounced than that of local students, who may have broader support networks and more stable socioeconomic safety nets within their native country. This finding suggests that societal contribution and quality are universal motivators, but practical factors like job security and market demand are particularly significant for certain subgroups.

Additionally, students born outside Qatar were less interested in patient-centric roles, potentially due to cultural or experiential differences. This result may be explained by expatriates feeling less culturally competent or comfortable interacting in healthcare settings because differences in language, customs, or healthcare beliefs and practices are prevalent in Qatar [33]. Cultural competence is crucial for patient-centered care, and a perceived lack of competence might deter students from pursuing patient-interacting roles [34]. Understanding these differences is crucial for educational institutions and healthcare providers seeking to foster greater inclusivity and support for all students, regardless of their background.

Gender disparities also emerged, with male students perceiving greater social influence on their career choices, likely due to cultural pressures emphasizing traditional gender roles and familial expectations. This aligns with previous studies indicating that males are more affected by social norms than females are [35]. This highlights the role of cultural dynamics in shaping gender-specific career motivations and choices in healthcare.

Students from higher-income families (above QR10,000/month) placed more importance on job quality than their middle-income peers did. This suggests that socioeconomic status plays a crucial role in shaping career priorities. This result is consistent with previous research indicating that individuals with lower incomes tend to make career decisions that offer quicker returns and smaller investments, whereas those with higher incomes seek courses with greater entrance difficulty [36]. The emphasis on quality among higher-income students reflects their ability to consider the intrinsic and long-term benefits of a career, which aligns with Herzberg’s theory, where individuals focus on self-actualization and quality once basic needs are met [37].

### Challenges and facilitators for healthcare education in Qatar

The study revealed that 43% of the students experienced significant stress due to heavy academic demands, including extensive coursework, clinical rotations, and high expectations. This finding is consistent with the literature indicating that healthcare students face higher levels of academic stress than their peers do in other fields [38]. The logistical challenges, such as long commutes and financial burdens, further exacerbate stress, reducing time for study and self-care. This finding aligns with previous research showing that long commuting times are associated with increased stress and lower academic performance among students [39]. Financial difficulties are also a well-documented barrier to academic success in healthcare education [40].

Despite these challenges, facilitators like emotional and financial support from family and friends are crucial in helping students successfully navigate their healthcare education. This finding is supported by research that emphasizes the role of social support in buffering the effects of academic stress and improving mental health outcomes among students [41]. Peer support has a significant influence on students’ academic engagement. For example, nursing students who perceived high levels of peer support also exhibited higher levels of academic engagement [42]. This correlation suggests that peer support can enhance students’ commitment to their studies and improve academic outcomes [43].

Institutional support, including accessible academic resources and engaged faculty, is essential for student retention and success [44]. Additionally, the use of effective time management strategies and external learning resources, such as YouTube and practice exams, indicates that students are actively seeking ways to enhance their learning experience and cope with academic demands. The reliance on digital tools for supplementary learning reflects the growing importance of technology in education. These tools offer flexible, personalized learning opportunities [45].

### International perspective

This study’s findings align with international research on healthcare students’ career choices, particularly in identifying intrinsic motivations such as altruism, societal contribution, and extrinsic factors such as job security as key factors. Similar studies from various global regions, including Western countries and the Middle East, report comparable drivers in healthcare career decision-making [1, 18]. In particular, research from the GCC region, such as Saudi Arabia and the UAE, has shown that job security and social status play significant roles in healthcare career choices, reflecting the strong cultural influence on career decisions [4, 46]. The challenges our participants faced, including academic pressures, financial burdens, and balancing family expectations, mirror global trends in healthcare education [47]. These challenges are especially pronounced in the GCC context, where cultural and socioeconomic factors—such as family influence and the importance of expatriate status—play a significant role in shaping the decision-making process [48].

### Strengths and limitations

A key strength of this research is its mixed-method survey design, which provides a comprehensive understanding of the factors influencing healthcare students’ career choices in Qatar. This mixed-method approach enables the study to quantify the facilitators and challenges encountered by students while also exploring deeper contextual reasons that underlie these factors. The collection of detailed demographic data allows for an analysis of how various variables, such as age, gender, nationality, and income, affect career choices. However, this study has several limitations. It relies on data from a single site, limiting the generalizability of findings to other contexts within Qatar or the broader region. Additionally, the cross-sectional design does not facilitate the investigation of changes in career motivations and challenges throughout students’ educational experiences. Furthermore, students from different healthcare disciplines may have varying expectations and priorities regarding career choice factors, which could influence the results and reduce the ability to generalize findings across all healthcare specialties. Finally, a potential limitation of this study is that the phrasing of Q2 and Q3 in the open-ended questions may have guided participants toward certain themes, potentially influencing the results.

### Implications

The findings of this study have important implications for healthcare education in Qatar. By identifying key factors influencing healthcare career choices, such as intrinsic motivations and job security, educational institutions can enhance these aspects to attract and retain students in healthcare programs. Practical experiences and highlighting the societal impact of healthcare professionals could increase students’ intrinsic motivation. The findings underscore the need for tailored support systems to address the challenges faced by students who are born outside Qatar, particularly in terms of cultural competence and job security. Policymakers should consider these insights to create a more inclusive and supportive educational environment, which is essential for building a sustainable and culturally competent healthcare workforce in Qatar. Future studies should explore individual healthcare disciplines to generate specific insights into unique career motivations and challenges. Longitudinal studies could also offer valuable insights into how these motivations and challenges evolve throughout students’ educational journeys, allowing for the development of tailored support strategies that adapt to their changing needs over time.

## Conclusion

This study highlights the complex interplay between personal motivation, economic factors, and educational challenges in shaping the career paths of healthcare students in Qatar. These insights are pivotal for policymakers and educational institutions aiming to refine healthcare education to better support students’ needs and aspirations. Addressing both intrinsic motivations and extrinsic challenges is essential to fostering student success. By implementing tailored support strategies, stakeholders can better support healthcare students in achieving their professional and personal goals, enhance student retention, and contribute to the development of a sustainable and culturally competent healthcare workforce capable of meeting Qatar’s evolving healthcare demands.

## Electronic Supplementary Material

Below is the link to the electronic supplementary material.

Supplementary Material 1

## Data Availability

The data supporting the findings of this study are available from the first author upon reasonaple request.
