# Supplementary material for: From decision to destination: factors influencing healthcare students’ career paths in Qatar
Source: BMC Med Educ. 2024 Dec 23;24:1522. doi: 10.1186/s12909-024-06527-3 (PMC11668116; doi:10.1186/s12909-024-06527-3)
Supplement: Supplementary file 1 — Supplementary Material 1 [file 12909_2024_6527_MOESM1_ESM.docx]

**HCC-Scale**

# Factor 1: Contribution to society

This health specialty allows me to offer service to society

This health specialty makes a worthwhile social health contribution

| 1 | 2 | 3 | 4 | 5 |
| --- | --- | --- | --- | --- |
| Not at all |  |  |  | Extremely |

| 1 | 2 | 3 | 4 | 5 |
| --- | --- | --- | --- | --- |
| Not at all |  |  |  | Extremely |

This health specialty will allow me to benefit the socially needy people

| 1 | 2 | 3 | 4 | 5 |
| --- | --- | --- | --- | --- |
| Not at all |  |  |  | Extremely |

This health specialty enables me to ‘give back’ to society

| 1 | 2 | 3 | 4 | 5 |
| --- | --- | --- | --- | --- |
| Not at all |  |  |  | Extremely |

This health specialty allows me to have an impact on people health

| 1 | 2 | 3 | 4 | 5 |
| --- | --- | --- | --- | --- |
| Not at all |  |  |  | Extremely |

# Factor 2: Social status

I think people working in this health specialty feel appreciated by society

| 1 | 2 | 3 | 4 | 5 |
| --- | --- | --- | --- | --- |
| Not at all |  |  |  | Extremely |

I think this health specialty is a well-respected career

| 1 | 2 | 3 | 4 | 5 |
| --- | --- | --- | --- | --- |
| Not at all |  |  |  | Extremely |

I think people working in this health specialty feel their occupation has high social rank

| 1 | 2 | 3 | 4 | 5 |
| --- | --- | --- | --- | --- |
| Not at all |  |  |  | Extremely |

I believe this health specialty is perceived as professionals

| 1 | 2 | 3 | 4 | 5 |
| --- | --- | --- | --- | --- |
| Not at all |  |  |  | Extremely |

I believe this health specialty is perceived as a high-status occupation

# Factor 3: Career value and Perceived abilities

This health specialty suited my abilities

| 1 | 2 | 3 | 4 | 5 |
| --- | --- | --- | --- | --- |
| Not at all |  |  |  | Extremely |

I have good skills specific for this health specialty

| 1 | 2 | 3 | 4 | 5 |
| --- | --- | --- | --- | --- |
| Not at all |  |  |  | Extremely |

I have the qualities needed to succeed in this health specialty

| 1 | 2 | 3 | 4 | 5 |
| --- | --- | --- | --- | --- |
| Not at all |  |  |  | Extremely |

I am interested in this health specialty

| 1 | 2 | 3 | 4 | 5 |
| --- | --- | --- | --- | --- |
| Not at all |  |  |  | Extremely |

I’ve always wanted to select this health specialty

| 1 | 2 | 3 | 4 | 5 |
| --- | --- | --- | --- | --- |
| Not at all |  |  |  | Extremely |

# Factor 4: Work with patients

I want to work in patient-centered environment I like working with patient of all ages

| 1 | 2 | 3 | 4 | 5 |
| --- | --- | --- | --- | --- |
| Not at all |  |  |  | Extremely |

| 1 | 2 | 3 | 4 | 5 |
| --- | --- | --- | --- | --- |
| Not at all |  |  |  | Extremely |

I want a job that involves working with patients of all ages

| 1 | 2 | 3 | 4 | 5 |
| --- | --- | --- | --- | --- |
| Not at all |  |  |  | Extremely |

# Factor 5: Satisfaction with choice

I’m satisfied with my selection of my current health specialty

| 1 | 2 | 3 | 4 | 5 |
| --- | --- | --- | --- | --- |
| Not at all |  |  |  | Extremely |

I carefully thought before selecting my current health specialty

| 1 | 2 | 3 | 4 | 5 |
| --- | --- | --- | --- | --- |
| Not at all |  |  |  | Extremely |

I’m happy with my decision about selecting this current health specialty

| 1 | 2 | 3 | 4 | 5 |
| --- | --- | --- | --- | --- |
| Not at all |  |  |  | Extremely |

# Factor 6: job Security

This health specialty provides a reliable income This health specialty will offer a steady career path This health specialty will provide me a secure job This health specialty is well paid

| 1 | 2 | 3 | 4 | 5 |
| --- | --- | --- | --- | --- |
| Not at all |  |  |  | Extremely |

| 1 | 2 | 3 | 4 | 5 |
| --- | --- | --- | --- | --- |
| Not at all |  |  |  | Extremely |

| 1 | 2 | 3 | 4 | 5 |
| --- | --- | --- | --- | --- |
| Not at all |  |  |  | Extremely |

| 1 | 2 | 3 | 4 | 5 |
| --- | --- | --- | --- | --- |
| Not at all |  |  |  | Extremely |

People working in this health specialty earns a good salary

| 1 | 2 | 3 | 4 | 5 |
| --- | --- | --- | --- | --- |
| Not at all |  |  |  | Extremely |

# Factor 7: Prior experiences

In this health specialty, I have seen some good role-models I have had positive health care experience

| 1 | 2 | 3 | 4 | 5 |
| --- | --- | --- | --- | --- |
| Not at all |  |  |  | Extremely |

| 1 | 2 | 3 | 4 | 5 |
| --- | --- | --- | --- | --- |
| Not at all |  |  |  | Extremely |

I have been amazed by some inspirational people at this health specialty

| 1 | 2 | 3 | 4 | 5 |
| --- | --- | --- | --- | --- |
| Not at all |  |  |  | Extremely |

I think this health specialty requires high levels of expert knowledge

| 1 | 2 | 3 | 4 | 5 |
| --- | --- | --- | --- | --- |
| Not at all |  |  |  | Extremely |

I think this health specialty need high levels of procedural knowledge

| 1 | 2 | 3 | 4 | 5 |
| --- | --- | --- | --- | --- |
| Not at all |  |  |  | Extremely |

I think this health specialty requires high level of specialized knowledge

| 1 | 2 | 3 | 4 | 5 |
| --- | --- | --- | --- | --- |
| Not at all |  |  |  | Extremely |

# Factor 8: Qualities

I think this health specialty requires high levels of expert

| 1 | 2 | 3 | 4 | 5 |
| --- | --- | --- | --- | --- |
| Not at all |  |  |  | Extremely |

| 1 | 2 | 3 | 4 | 5 |
| --- | --- | --- | --- | --- |
| Not at all |  |  |  | Extremely |

| 1 | 2 | 3 | 4 | 5 |
| --- | --- | --- | --- | --- |
| Not at all |  |  |  | Extremely |

Knowledge

I think this health specialty need high levels of procedural knowledge

I think this health specialty requires high level of

specialized knowledge

# Factor 9: Social influences

My family think I should study this health specialty People I’ve met think I should study this health specialty My friends encourage me to study this health specialty

| 1 | 2 | 3 | 4 | 5 |
| --- | --- | --- | --- | --- |
| Not at all |  |  |  | Extremely |

| 1 | 2 | 3 | 4 | 5 |
| --- | --- | --- | --- | --- |
| Not at all |  |  |  | Extremely |

| 1 | 2 | 3 | 4 | 5 |
| --- | --- | --- | --- | --- |
| Not at all |  |  |  | Extremely |

# Factor 10: Fallback career

I chose this health specialty as a last-resort career I was not accepted into my first-choice career

| 1 | 2 | 3 | 4 | 5 |
| --- | --- | --- | --- | --- |
| Not at all |  |  |  | Extremely |

| 1 | 2 | 3 | 4 | 5 |
| --- | --- | --- | --- | --- |
| Not at all |  |  |  | Extremely |

I was unsure of what health specialty I wanted

| 1 | 2 | 3 | 4 | 5 |
| --- | --- | --- | --- | --- |
| Not at all |  |  |  | Extremely |

# Factor 11: Difficulty

I think this health specialty is hard

| 1 | 2 | 3 | 4 | 5 |
| --- | --- | --- | --- | --- |
| Not at all |  |  |  | Extremely |

I think people working in this health specialty have heavy workload

| 1 | 2 | 3 | 4 | 5 |
| --- | --- | --- | --- | --- |
| Not at all |  |  |  | Extremely |

# Factor 12: Social dissuasion (socialization)

I was encouraged to select other than this health specialty

| 1 | 2 | 3 | 4 | 5 |
| --- | --- | --- | --- | --- |
| Not at all |  |  |  | Extremely |

I was told by others that selecting this health specialty was not a good decision

| 1 | 2 | 3 | 4 | 5 |
| --- | --- | --- | --- | --- |
| Not at all |  |  |  | Extremely |

I was influenced to consider other career than this health specialty

| 1 | 2 | 3 | 4 | 5 |
| --- | --- | --- | --- | --- |
| Not at all |  |  |  | Extremely |

# Demographic data sheet

- **Age: _________Years**
- **Gender:**
  - Male
  - Female
- **Sponsorship:**
  - Family sponsorship
  - Private sponsorship
  - Government sponsorship
- **Income/family income:**
  - Less than QR 5,000
  - Between QR 5,001-10,000
  - More than 10,000
- **Place of Birth**
  - Inside Qatar
  - Outside Qatar
- **Where did you complete your high school?**
  - Inside Qatar
  - In the GCC countries other than Qatar
  - In the Arab countries other than GCC countries
  - In Asian countries other than Arab countries
  - In Europe, North America or Australia
  - In Africa
- **Healthcare specialty**
  - Nursing
  - Pharmacy
  - Medical radiology
  - Midwifery
  - Respiratory Therapy
  - Dental Hygiene
- **Please indicate the total number of years you have been enrolled as a student at this university:**
  - Less than 1 year
  - 1-2 years
  - 3-4 years
  - More than 4 years
- **Your GPA at the high school**
  - Less than 2 (less than 60%)
  - 2 to 3 (60% to less than 70%)
  - 3.1 to 4 (70% or above)

# Open ended questions

- If you could go back in time, would you still choose your profession? why?
- What are the challenges faced in terms of academic demands, personal life, social pressures, or financial burdens during your study?
- What are the facilitators that helped you to overcome the challenges during your study?
